# Conditional Random Field (CRF)-Boosting: Constructing a Robust Online Hybrid Boosting Multiple Object Tracker Facilitated by CRF Learning

**DOI:** 10.3390/s17030617

**Published:** 2017-03-17

**Authors:** Ehwa Yang, Jeonghwan Gwak, Moongu Jeon

**Affiliations:** School of Electrical Engineering and Computer Science, Gwangju Institute of Science and Technology, Gwangju 61005, Korea; ehwa@gist.ac.kr (E.Y.); james.han.gwak@gmail.com (J.G.)

**Keywords:** visual sensors, multiple object tracking, data association, conditional random fields, boosting algorithms, hybrid approaches

## Abstract

Due to the reasonably acceptable performance of state-of-the-art object detectors, tracking-by-detection is a standard strategy for visual multi-object tracking (MOT). In particular, online MOT is more demanding due to its diverse applications in time-critical situations. A main issue of realizing online MOT is how to associate noisy object detection results on a new frame with previously being tracked objects. In this work, we propose a multi-object tracker method called CRF-boosting which utilizes a hybrid data association method based on online hybrid boosting facilitated by a conditional random field (CRF) for establishing online MOT. For data association, learned CRF is used to generate reliable low-level tracklets and then these are used as the input of the hybrid boosting. To do so, while existing data association methods based on boosting algorithms have the necessity of training data having ground truth information to improve robustness, CRF-boosting ensures sufficient robustness without such information due to the synergetic cascaded learning procedure. Further, a hierarchical feature association framework is adopted to further improve MOT accuracy. From experimental results on public datasets, we could conclude that the benefit of proposed hybrid approach compared to the other competitive MOT systems is noticeable.

## 1. Introduction

Multiple object tracking (MOT) [1,2] is one of the most important and hectic areas in the field of computer vision research, and recent advances on detection and tracking of multiple objects have led to its application to diverse practical problems such as bio-medical imaging, visual surveillance systems and augmented reality. The main tasks of establishing MOT systems are to extract positions of objects, to generate the trajectories of each individual object, and to maintain the identity of each object, even for crowded environments. There are several issues that increase MOT complexity such as imprecise and noisy detections, occlusions by the other objects or background, and dynamic interactions among objects.

Due to the success in developing robust object detectors [3,4,5], many recent studies on MOT adopt tracking-by-detection approaches [6,7,8,9,10,11,12,13,14,15,16,17,18,19,20], where the key research topic is data association to link object detections or tracklets (i.e., track fragments) in a sequence of frames for assembling the final trajectories of the objects. Such MOT systems based on data association consist of two main components: (1) *a tracklet affinity model* measuring the likelihood (or linking probability) that two detection responses or tracklets belong to the same target; and (2) *a global optimization framework* determining which detection responses or tracklets should be linked based on the affinity measurement, which is commonly formulated as a maximum a posteriori problem.

Although many methods have been proposed to develop global optimization frameworks based on linear programming [6], min-cost flow algorithm [7] and Hungarian algorithm [8], relatively less effort has been devoted to improving the affinity model. Simple affinity models widely adopted for efficiency purposes are mostly based on straightforward parametric models (e.g., Gaussian distributions for object location changes and distance between color histograms for object appearance affinity measurement). Moreover, in many cases, the model parameters and the relative emphases of different cues are determined depending on prior knowledge or human observation of the data. When environmental changes or different cues (e.g., appearance, motion, and context information) are combined into one affinity model, it is almost impossible to tune the model manually.

To overcome such difficulties, we propose a hybrid data association algorithm combining conditional random field (CRF) [21,22] and online hybrid boosting for building robust MOT. Existing data association approaches adopting different machine learning techniques such as boosting need training sets with ground truth information [23,24] for higher accuracy. While *rank boost* [23] achieves better performance than *binary boost* [23], it is very difficult to design an online algorithm for this because of its ranking concept. CRF is a powerful model adopted in many computer vision research fields, but not widely utilized in data association for MOT. In our work, with the aim of designing an online MOT system, we incorporate CRF, which enables low-level data association into a hybrid boosting-based data association approach with a ranking concept. Specifically, we represent the association of detection responses between two frames as a graph for CRF, and design an online algorithm by applying the results of CRF-based pairwise similarity matching to build the training data. Finally, the CRF learning output is used for the input to the hybrid boosting algorithm that learns tracklet affinity models. To this end, the contributions of this work are as follows:

- A robust hybrid data association is proposed by cascading robust CRF-based pairwise similarity matching and online hybrid boosting.
- A hierarchical feature association framework is adopted to improve the accuracy.
- A fully automated online MOT method called CRF-boosting is established.

The rest of this paper is organized as follows: the preliminaries of this work, CRF and boosting approaches, are described in Section 2. Section 3 describes the details of the proposed hybrid MOT approach. The experimental results and analysis are given in Section 4. Finally, the conclusions and future work are given in Section 5.

## 2. Related Work

One key issue in MOT is how to distinguish targets from background and other objects. To do this, researchers usually try to find or learn proper appearance models which have the capabilities of identifying one target from among all other objects or background. Also, to perform effective tracklets associations, data association frameworks have been widely studied. Most of the MOT methods usually take the tracking-by-detection approaches [6,7,8,9,10,11,12,13,14,15,16,17,18,19,20,25,26,27,28,29,30,31,32,33,34,35] and can be classified into two categories: (1) MOT utilizing past and current frames for association decisions (e.g., [34,35]); and (2) MOT using all the frames, including past, current, and future frames (e.g., [7,8,9,24,36]). The former usually adopts a particle filtering framework based on detection responses, and it is more suitable for time-critical applications and systems because it does not require future frames. However, it is very vulnerable to noisy observations and long-term occlusions of targets. To obtain further improved results, the latter uses all the frames and adopts global optimization. The tracking-by-detection-based MOT methods usually associate detection responses obtained from a pre-trained detector into tracklets progressively and finally construct trajectories for all targets. Appearance models, whether pre-trained or online learned, are commonly adopted to distinguish targets. In addition, motion models can be also adopted to predict the feasible position of objects in the future frames, which reduces the search space. The appearance and motion models may be optimized, but for differentiating all targets each other, there are still some challenges such as: (1) similar appearance of targets; (2) complex interactions among objects; (3) frequent occlusions; (4) different size of targets, and (5) initialization and termination of tracks.

Among the related works, we mainly focus on reviewing the closely related works on boosting-based MOT [9,24,27,37] and CRF-based MOT [33,38]. Boosting-based MOT is easier to implement than CRF-based MOT, and boosting can be used in combination with different learning algorithms to improve its performance. In boosting-based MOT, most studies focus on improving the robustness and effectiveness of appearance models which can be used as distinctive feature information. In contrast, studies on CRF-based MOT have usually focused on data association to generate final trajectories. In Li et al. [24], HybridBoost was used to learn an appearance model which is integrated in a hierarchical data association framework [39] to progressively grow tracklets. In Yang et al. [9] devised a part-based appearance modelling and grouping-based data association framework to alleviate the problems of frequent occlusions and similar appearances among objects. A boosting algorithm was used to learn a part-based appearance model. In Kuo et al. [37], an online learning approach to build a discriminative appearance model was proposed. The AdaBoost algorithm is used to combine effective image descriptors and their corresponding similarity measurements. To make online learning possible, positive and negative training samples are obtained from the results of short but reliable tracklets using a dual-threshold method [39]. Bae and Yoon [27] proposed online MOT based on tracklet confidence and online discriminative appearance learning. Effective tracklets are obtained by sequentially linking detections/tracklets using local and global association according to their confidence levels, and incremental linear discriminant analysis [32] is used for online discriminative appearance model learning. Yang et al. [33] proposed a CRF model to consider both tracklet affinities and dependences among tracklets, and to transform the problem of MOT into an energy minimization task. In Yang and Nevatia [38], an online learned CRF model was used to generate final trajectories. For online learning, low-level tracklets are required and are generated by simply using color or location information between two consecutive frames. However, in many cases, this is not practical because it can increase association errors under noisy observation conditions.

## 3. Background

In this section, the two key elements, CRF and hybrid boosting methods, which are used to build an effective tracklet affinity model in Section 3, are explained in detail.

### 3.1. Conditional Random Fields

CRFs are discriminative undirected probabilistic graphical models developed for labeling/segmenting structural and sequential data [21,40], and it is shown in [41] that they are competent in modelling spatial relationships. We can define conditional distribution p(x|z) over the hidden variables *x* given observation *z* where nodes $x=<x_{1},x_{2},\ldots ,x_{n}>$ represents hidden states and nodes $z=<z_{1},z_{2},\ldots ,z_{n}>$ indicates data. Using the nodes *x_i_* and their connectivity structure represented by undirected edges, we define the conditional distribution p(x|z) over *x*. Suppose *C* is the set of cliques which are fully connected subsets in the graph of a CRF, the CRF can factorize the conditional distribution into a product of pairwise clique potentials $\phi_{c}(z,x_{c})$, where every c∈C is a clique in the graph, *x_c_* is the variable of the hidden node and *z* is the observation in the clique. By clique potentials, the conditional distribution over hidden states is written as:

(1) $$p(x|z)=\frac{1}{Z(z)}\prod_{c\in C}\phi_{c}(z,x_{c}),$$

where $Z(z)=\sum_{x}\prod_{c\in C}\phi_{c}(z,x_{c})$ is the normalizing partition function. Also, $\phi_{c}(z,x_{c})$ is described by log-linear combinations of feature functions *f_c_* as follows:

(2) $$\phi_{c}(z,x_{c})=\exp(w_{c}^{T}\cdot f_{c}(z,x_{c})),$$

where $w_{c}^{T}$ is a weight vector, and $f_{c}(z,x_{c})$ is a feature function. Then, (1) can be rewritten as:

(3) $$p(x|z)=\frac{1}{Z(z)}\exp(\sum_{c\in C}w_{c}^{T}\cdot f_{c}(z,x_{c})).$$

The weights of the feature functions in (3) are determined by the CRF parameter learning. CRF learns the weights discriminatively through maximizing the conditional likelihood of labeled training data. We can find the global optimum of (3) using a numerical gradient method, but it is very inefficient because the inference procedure of the optimization should be executed at each iteration. Thus, we adopt the method of maximizing the pseudo-likelihood of the training data and it is given by the sum of local likelihoods $\left(x_{i}|MB(x_{i})\right)$, where $MB(x_{i})$ is the *x_i_*’s Markov blanket indicating the set of the immediate neighbors of *x_i_* in the CRF graph [42]. The optimization is performed by minimizing:

(4) $$L(w)=-\sum_{i=1}^{n}\log p(x_{i}|MB(x_{i}),w)+\frac{{\left(w-\tilde{w}\right)}^{T}(w-\tilde{w})}{2\sigma^{2}},$$

where the rightmost term represents a Gaussian shrinkage prior with mean $\tilde{w}$ and variance $\sigma^{2}$. We use unconstrained L-BFGS [36] as a gradient descent method to optimize (4). Then, at the inference stage using a new test data, the learned CRF estimate the most likely configuration of all hidden variables ***x*** using belief propagation [40].

### 3.2. Hybrid Boosting

Boosting has been successfully used in a variety of machine learning tasks and widely applied to computer vision tasks as well. In this section, for learning an appearance affinity model, we introduce a hybrid boosting algorithm having the property of both a ranking function and a binary classifier.

A ranking problem includes an instance space *X* with a ranking function *H* that defines a linear ordering of instances in *X*. *H* takes the form of H:X→R. Proposed by Freund et al. [23], rank boost is an algorithm invented for this purpose. In rank boost, a set of instance pairs $R=\left\{<x_{i},x_{j}>|x_{i},x_{j}\in X\right\}$ constitute training data, where *x_j_* should be ranked higher than *x_i_*, $H(x_{j})>H(x_{i})$. The aim is finding such *H* that describes the ranking over *X*.

We can map the ranking problem onto the data association problem. We define instance *X* to be *T* × *T* where *T* is the set of tracklets to be possibly associated. For example, given tracklets $T_{1},T_{2},T_{3},T_{4}\in T$, if *T*_1_ and *T*_3_ are the real trajectory that should be correctly linked, then the ranking must be $H(<T_{1},T_{3}>)>H(<T_{1},T_{2}>)$ and $H(<T_{1},T_{3}>)>H(<T_{1},T_{4}>)$. When *T^t^* is the terminating tracklet of a target trajectory, to prevent associating *T^t^* to any other tracklet *T^c^*, it is defined as $H(<T^{t},T^{c}>)<\zeta ,\;\forall T^{c}\in T$ where ζ is a rejection threshold. Also, objects in different tracklets in a frame (i.e., at the same time) cannot be the same target. In these cases, it becomes the problem of both ranking and binary classification to define an impossible association link.

To resolve the problem, in the hybrid boosting algorithm, the training set is composed of a ranking sample set *R* and a binary sample set *B*. The ranking sample set is denoted by:

(5) $$R=\left\{(x_{i,0},x_{i,1})|x_{i,0}\in X,x_{i,1}\in X\right\},$$

where each *x_i_*_,0_ and *x_i_*_,1_ represents a pair of tracklets, and $(x_{i,0},x_{i,1})\in R$ means that the association of *x_i_*_,1_ is ranked higher than *x_i_*_,0_. The binary sample set is denoted by:

(6) $$B=\{(x_{j},y_{j})|x_{j}\in X,y_{j}\in \{-1,1\}\},$$

where *y_j_* = 1 indicates the corresponding *x_j_* should be associated at any time, and *y_j_* = −1 means the corresponding *x_j_* should not be associated. A loss function for the hybrid boosting is defined as a linear combination of the ranking loss function and the binary classification loss function given as:

(7) $$Z=\beta \sum_{(x_{i,0},x_{i,1})\in R}w_{0}(x_{i,0},x_{i,1})\exp(H(x_{i,0})-H(x_{i,1}))+(1-\beta )\sum_{(x_{j},y_{j})\in B}w_{0}(x_{j},y_{j})\exp(-y_{j}H(x_{j})),$$

where *β* is a constant coefficient and *ω*_0_ is the initial weight function. In the boosting algorithm, to find *H*(*x*), we need to minimize *Z*, and *H* can be obtained by adding new weak ranking classifiers sequentially. Therefore, (7) can be written using weak ranking classifier h(t):X→R and its weight *α_t_* as follows:

(8) $$Z=\beta \sum_{(x_{i,0},x_{i,1})\in R}w_{0}(x_{i,0},x_{i,1})\exp(\alpha_{t}(h_{t}(x_{i,0})-h_{t}(x_{i,1})))+(1-\beta )\sum_{(x_{j},y_{j})\in B}w_{0}(x_{j},y_{j})\exp(-y_{j}\alpha_{t}h_{t}(x_{j})),$$

Finally, the final strong ranking classifier is the weighted combination of the selected weak ranking classifiers as follows:

(9) $$H(x)=\sum_{t=1}^{n}\alpha_{t}h_{t}(x),$$

where *n* is the number of boosting rounds. Attributed to the loss function *Z*, *H*(*x*) contains the advantage of both a ranking classifier and a binary classifier.

## 4. Proposed Approach: CRF-Boosting

In this section, based on the CRF and hybrid boosting discussed in Section 3, we demonstrate how to design a robust online MOT system called CRF-boosting.

### 4.1. Overall Procedure

**Figure 1:**
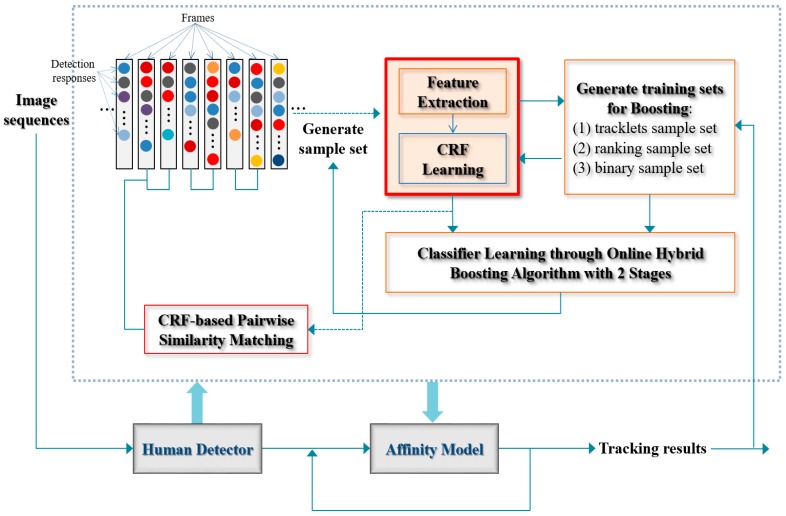
Schematics of the proposed MOT system.

For tracking multiple objects robustly under difficult conditions such as with noisy or missed detections, many boosting-based data association methods have used training data with ground truth (GT) information or the like. In many cases, due to the impracticality and inconvenience of obtaining training data with accurate GT information in different situations, offline learning of an affinity model was commonly adopted. However, in such a way, it is very difficult to implement robust online MOT with real-time processing capability. To overcome this drawback, in this work, we generate a CRF model for intermittent temporary tracklet association between two consecutive frames, and the results (i.e., those with selected good samples) from the CRF model are used as the training data for hybrid boosting to establish an online MOT system called CRF-boosting. In addition, based on hierarchical feature association through online hybrid boosting algorithm, detection responses are progressively linked into longer ones to form final tracking outcomes in an online manner. Figure 1 shows the overall schematics of the proposed system.

At the first step, as input data, detection responses are obtained from image sequences. In the hybrid boosting algorithm, we use not only ranking information, but also binary information, and thus it is very crucial to utilize accurate and reliable tracklet information in its training process. To do this, we use a learned CRF model [40] which can give the similarity information between objects in two consecutive frames. The construction of the CRF model is described in Section 4.2. The reliable short tracklets constructed by the CRF model are used as the input of the hybrid boosting-based data association algorithm that produces the final trajectory information. The details of the hybrid boosting are described in Section 4.3.

### 4.2. CRF Matching

**Figure 2:**
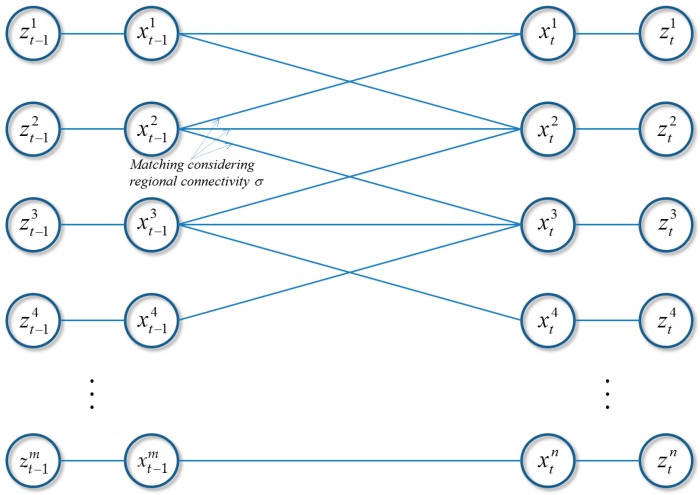
Graph of a CRF between frame *t* − 1 and frame *t.*

In CRF, intermittent temporary connections among detected objects between frames are made with the feature information of the objects. To find the links between two frames, we generate a CRF graph that contains hidden node $x_{t}^{i}$ indicating object *i* in frame *t*. In generating a graph of CRF, node $x_{t-1}^{i}$ is not connected with all nodes $x_{t}^{i}$ at the next frame *t*; Node $x_{t-1}^{i}$ is connected with $x_{t}^{i}$ within certain boundary *σ* from its position (i.e., only neighboring objects are connected) using regional (i.e., local proximity-based) connectivity assuming that the object is not moving suddenly far away between two consecutive frames. Here, we set the σ=2.5×hight of object i. Then, considering the local proximity, an efficient CRF model can be constructed. An example is given in Figure 2.

Node $z_{t}^{i}$ in Figure 2 corresponds to the local features (i.e., observation data) of hidden node $x_{t}^{i}$ (i.e., object *i*). In this work, we use the spatial distance [40] and visual appearance including color histogram [22] and covariance [37,43] as the features. Then, (3) in Section 3.1 expresses conditional distribution of the CRF, and the function of each feature for similarity measurement is defined as the differences of features among the objects. In this CRF, the feature function of spatial distance between object *j* in frame *t* − 1 and object *i* in frame *t* is defined as:

(10) $$f_{sd}(i,j,z_{t}^{i,sd},z_{t-1}^{j,sd})=\frac{{\left\|z_{t}^{i,sd}-z_{t-1}^{j,sd}\right\|}^{2}}{\sigma_{sd}^{2}},$$

where $z_{t}^{i,ch}$ is the position of individual points in *i*, $z_{t-1}^{j,sd}$ is the position of individual points in *j*, and *σ*^2^ is the variance of the distances in the training data. The feature function of color histogram is defined as:

(11) $$f_{ch}(i,j,z_{t}^{i,ch},z_{t-1}^{j,ch})=\frac{{\left\|z_{t}^{i,ch}-z_{t-1}^{j,ch}\right\|}^{2}}{\sigma_{ch}^{2}},$$

where $z_{t}^{i,ch}$ is the color histogram of *i*, $z_{t-1}^{j,sd}$ is the color histogram of *j*, and *σ*^2^ is the variance of the color histogram differences in the training data. Single channel histograms are concatenated to construct a single vector with 8 bins for each channel, resulting a 24-dimensional vector. Next, the feature function of covariance is computed by:

(12) $$f_{cov}(i,j,C_{i},C_{j})=\sqrt{\sum_{k=1}^{7}\ln^{2}\gamma_{k}(C_{i},C_{j})},$$

where ${\left\{\lambda_{k}(C_{i},C_{j})\right\}}_{k=1,\ldots ,7}$ are the generalized eigenvalues of *C_i_* and *C_j_* computed from $\lambda_{k}C_{i}x_{k}-C_{j}x_{k}=0$ where $x_{k}(\neq 0)$ are generalized eigenvectors; *C_i_* corresponds to the covariance matrix defined as:

(13) $$C_{i}=\frac{1}{P-1}\sum_{p=1}^{P}(z_{i,p}-\mu_{i}){\left(z_{i,p}-\mu_{i}\right)}^{T},$$

where *P* is the number of pixels in the region of *i*, denoted as $R_{i}$, $\mu_{i}$ is the pixel mean vector over $R_{i}$, *I* is the intensity of the pixel and $z_{i,p}$ is the vector consists of the first and second derivatives of $R_{i}$ at *p*-th pixel, which is given as:

(14) $$z_{i,p}={\left[\left|\frac{\partial I}{\partial x}\right|\;\left|\frac{\partial I}{\partial y}\right|\;\left|\frac{\partial^{2}I}{\partial x^{2}}\right|\;\left|\frac{\partial^{2}I}{\partial y^{2}}\right|\;\left|\frac{\partial^{2}I}{\partial xy}\right|\right]}^{T}.$$

Similar to [43], the image derivatives are computed using the filters ${\left[-1\;0\;1\right]}^{T}$ and ${\left[-1\;2\;-1\right]}^{T}$, resulting covariance of a region is a 9 × 9 matrix.

### 4.3. Composing Training Sets using CRF Matching Output

**Figure 3:**
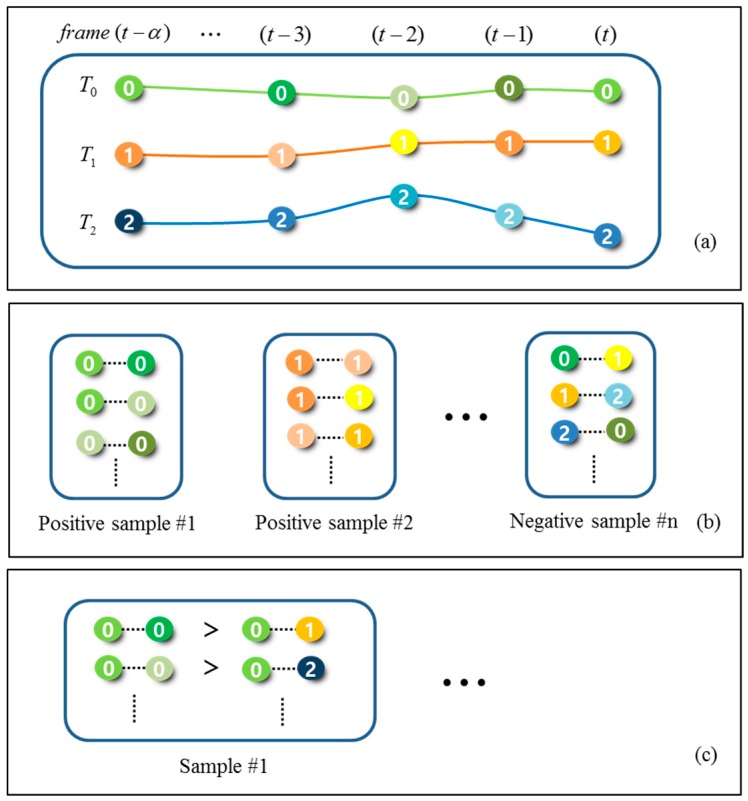
Training dataset: (**a**) example of tracklets; (**b**) composing binary training sets from (**a**); (**c**) composing ranking training sets from (**a**).

For learning a hybrid boosting algorithm in an online manner, we have to compose training sets automatically. In this work, the information of matched detection responses as a result of CRF matching (Section 4.2) in consecutive frames are employed for the purpose. The spatio-temporal distance information is used for composing training dataset. The training datasets are divided into the ranking dataset and the binary dataset, where each dataset consists of positive and negative datasets for learning the boosting algorithm. Then, we assume that each tracklet corresponds to an object and the targets at a frame (i.e., at the same time) constitutes the tracklets different from each other. That is, since it is trivial that the objects in different trajectories cannot be the same target, we use this spatio-temporal constraint for building the training data. In this way, using the reliable tracklets output of the CRF matching, we can construct the training dataset for the boosting algorithm. We used the ranking training set defined in (5) and the binary training set defined in (6). Figure 3 shows an example of constructing the training dataset.

### 4.4. Hybrid Boosting

Similar to [24], as shown in Table 1, 13 types of hierarchical features, representing length of tracklets (idx 1 to 3), appearance information of tracklets (idx 4, 5), frame gap information of tracklets (idx 6 to 9), and motion information of tracklets (idx 10, 11), are adopted in this work. The online hybrid boosting algorithm is given in Algorithm 1 (details was discussed in Section 3.2). In the boosting algorithm, each feature is a function f:x→R, which takes a pair of tracklets $x=<T_{i},T_{j}>$ as its input and outputs a real value. The weak ranking classifier is defined as:

(15) $$h(x)=\{\begin{array}{ll} +1 & if\;f(x)>\delta \\ -1 & othewise \end{array}.$$

**Figure 4:**
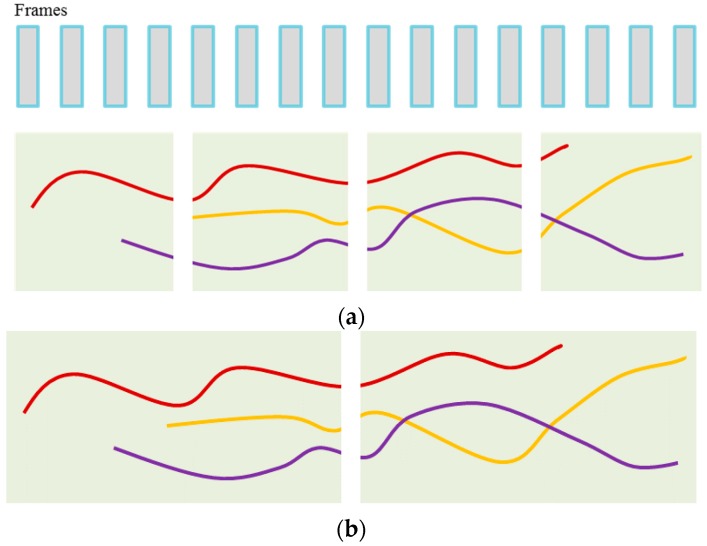
Two-stage training procedure: (**a**) 1st stage: Maximum length of tracklets is 1/4 of the whole image sequences for training; (**b**) 2nd stage: Maximum length of tracklets is 1/2 of whole image sequences for training.

As described in Figure 4, we design the boosting algorithm with two stages in its training procedure. In constructing trajectories, the two stage training procedures can help to exploit more accurate ranking information, e.g., by appearance affinity models with different poses in a trajectory, through considering different length of tracklets. For this, the maximum length in the first stage is defined as the 1/4 of the full training image sequences, and that in the second stage is 1/2 of the sequences. By training incrementally, we can obtain the more accurate tracklets information rather than utilizing all image sequences at once, which improves the MOT system robustness (i.e., capable of reducing tracking errors).

The procedure of the proposed CRF-boosting algorithm is given in Algorithm 2. In the proposed CRF-Boosting tracker, two-stage training is performed. As a result of CRF-based pairwise similarity matching, robust low-level tracklets are obtained and using them, ranking and binary classification samples are formed in an online manner. Then, a strong ranking classifier H(x) is learned using hybrid boosting in Algorithm 1. The CRF-boosting tracker using H(x) as the tracklet affinity model is then applied to generate the 1st stage association. The above procedures are repeated to establish the 2nd stage association. Finally, trajectories for all targets are constructed.
**Algorithm 1:** Online Hybrid Boosting Algorithm
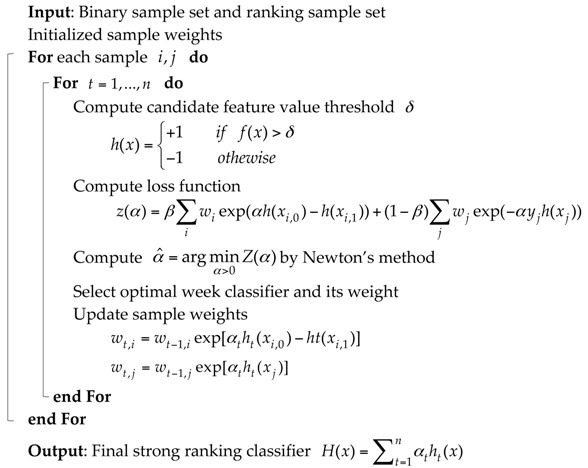

**Algorithm 2:** CRF-Boosting Tracker with the Two-Stage Training Procedure
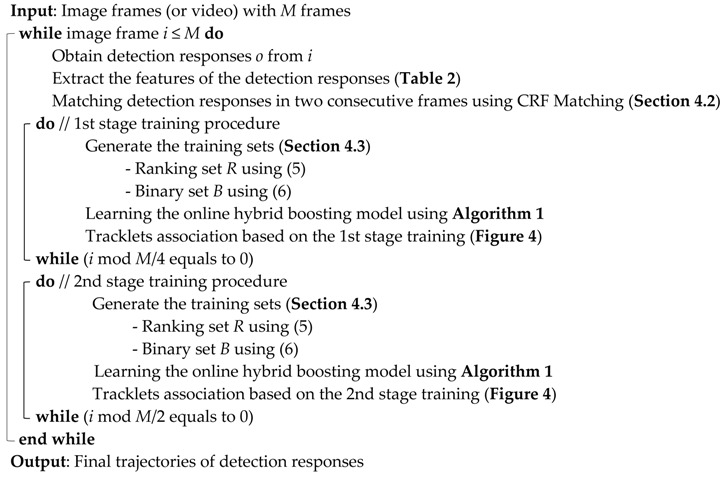


## 5. Experimental Results and Analysis

In this section, the experimental results, their analyses, and the experimental conclusions that can be drawn are discussed. We evaluate the effectiveness of our proposed MOT system with three widely used public surveillance datasets: CAVIAR [44], PETS2009 [45] and ETH [46]. The CAVIAR dataset contains 26 video sequences of corridor in a shopping mall taken by a single camera with frame size of 384 × 288 and frame rate of 25 FPS. The PETS2009 dataset include the “S2.L1” (sparsely crowded scenes), “S2.L2” (moderately crowded scenes), “S2.L3” (densely crowded scenes) videos taken by a multiple static camera with frame size of 768 × 576 pixels and frame rate of 25 FPS. The ETH dataset contains video sequences taken by a stereo forward-looking camera mounted on a moving children’s stroller on busy street scenes. The frame rate is 14 FPS and the image size is 640 × 480 pixels for the videos. We chose the “Bahnhof” and “Sunny day” sequences from the ETH dataset. The human detection results are the same as used in [37,38] and are provided by courtesy of authors of [22].

### 5.1. Evaulation Metrics

Following the metrics used in [24], we use the evaluation metrics described in Table 2. The better MOT performance is obtained for the *higher* values in RC and MT and for the *lower* values in FAF, ML, FRG and IDS. By the definitions, the total sum of MT, PT and ML should be 100%. In general, a higher value of PT is better, but if MOT improves MT by better association capability PT can be decreased because it can result in lesser partial trajectories. That is, PT depends on the tracklet association performance of MOT. Therefore, we exempt PT from the analyses of the experimental results, but it is remained in the resulting tables, Table 3, Table 4, Table 5 and Table 6, for the readers’ reference.

### 5.2. Experimental Results and Discussion

**Figure 5:**
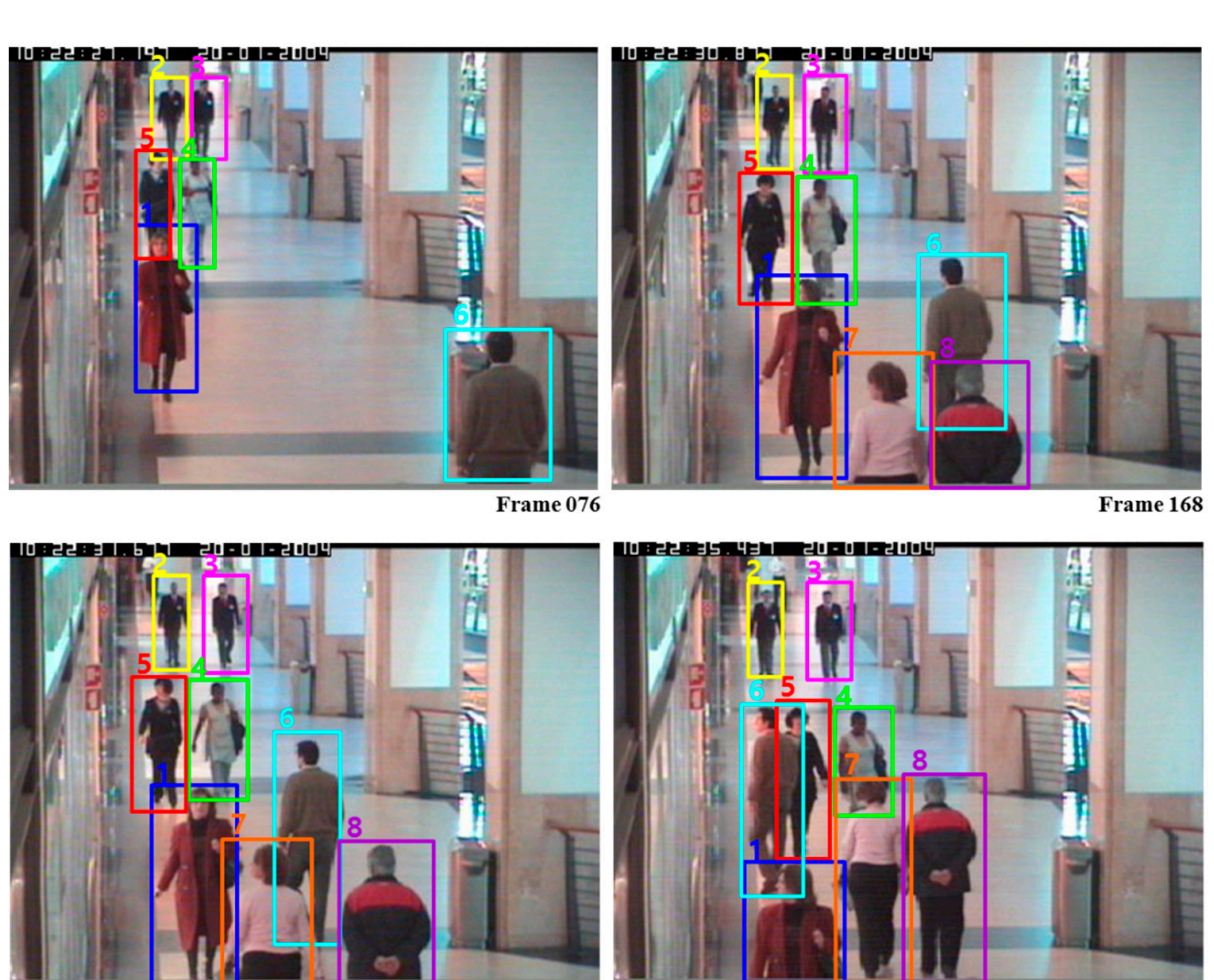
Tracking results of our system on CAVIAR.

*Results and Analysis on the CAVIAR dataset:* Wu and Nevatia [47] presented body-part detection based MOT in which a human are represented by four body parts including full-body, head-shoulder, torso and legs. Zhang et al. [7] introduced a min-cost flow network based data association framework with a non-overlap constraint on trajectories. Huang et al. [39] devised three-level hierarchical data association approach. At the low level, reliable short tracklets are obtained, and at the middle level, the Hungarian algorithm is applied to further associate the short tracklets. At the high level, using the computed tracklets, entries/exits and occlusions are estimated, and final trajectories are refined using them. Li et al. [24] proposed a HybridBoost algorithm for learning tracklet affinity models in which the problem of ranking and classification is jointly considered. Kuo et al. [37] proposed online learned discriminative appearance models (OLDAM) to enhance MOT accuracy through discriminative appearance modelling using an AdaBoost algorithm. Bak et al. [28] proposed an algorithm to learn discriminative appearance models based on a mean Riemannian covariance grid descriptor obtained from tracklets given by short-term tracking. Yang et al. [48] devised MOT by online nonlinear motion patterns learning and a multiple instance learning based on incrementally learned entry/exit map. Table 3 shows the comparison results of the proposed approach with the competing MOT methods on the CAVIAR dataset. From Table 3, it is obviously seen that the proposal could achieve the best performance than the others in terms of RC and PRCS, and generally good performance in terms of FAF, MT and IDS. The instances of the tracking results using CRF-Boosting MOT are shown in Figure 5.

*Results and Analysis on the PETS dataset:* Kuo et al. [22] proposed a Person Identity Recognition- based Multi-Person Tracking (PIRMPT) method where they used person recognition and divided reliable tracklets as query tracklets and gallery tracklets in which for each gallery tracklet a target-specific appearance-based affinity model is learned. 

**Figure 6:**
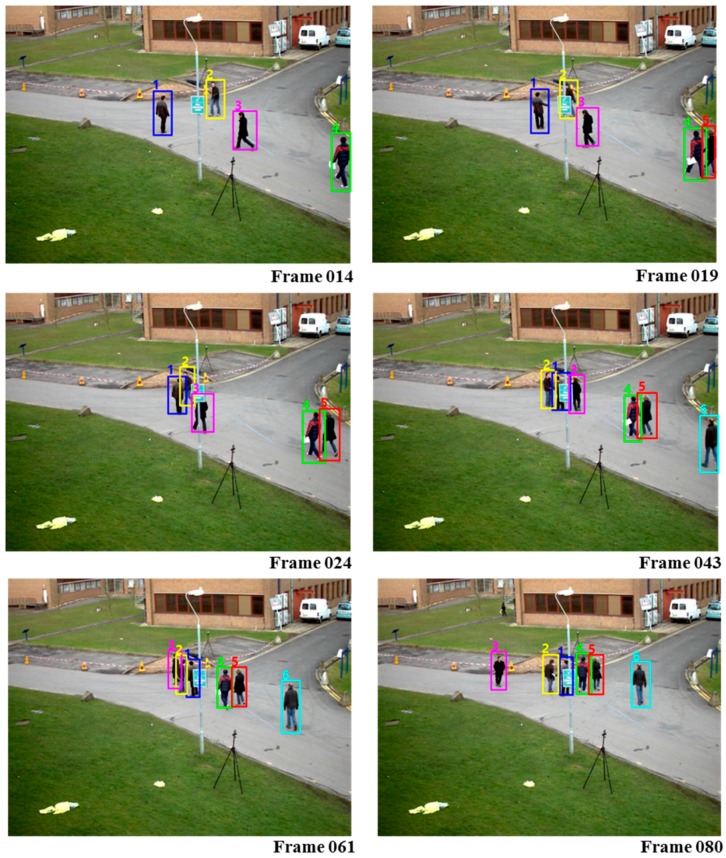
Tracking results of our system on PETS2009.

PIRMPT used the similar framework of OLDAM [37] in collecting training samples for learning online discriminative appearance models but it further improved by automatic learning of discriminative features obtained from the target-specific appearance information. From Table 4, compared to the other algorithms it can be seen that CRF-Boosting could obtain best performance in terms of ML and IDS and comparable performance in terms of PRCS and FRG. The instances of the tracking results using CRF-Boosting MOT are shown in Figure 6.

*Results and Analysis on the ETH dataset:* Kim et al. [49] proposed an online data association which is formulated as a bipartite matching and solved by structural support vector machines (S-SVM). In Bo and Nevatia [38], an online learned CRF model is used and MOT is formulated as an energy minimization problem where energy functions consists of a set of unary functions based on appearance and motion models to discriminate targets. 

**Figure 7:**
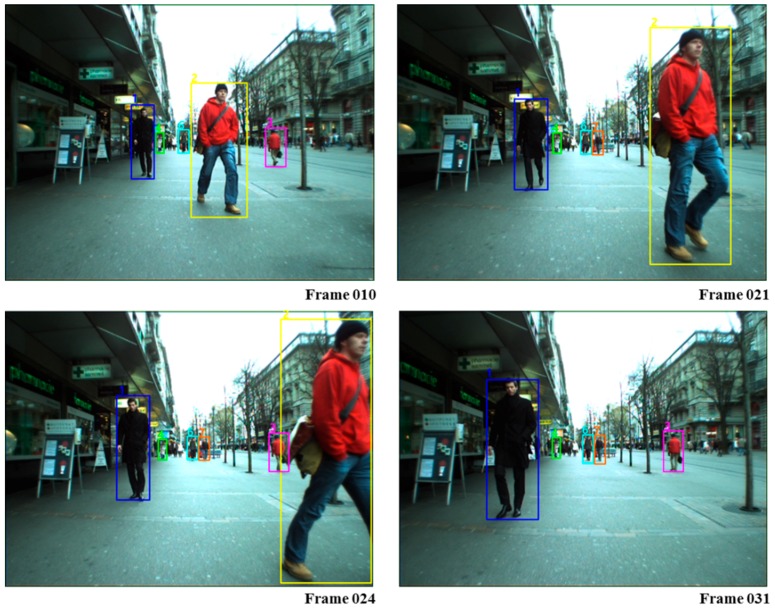
Tracking results of our system on ETH.

From Table 5, it can be trivially seen that the proposed method could outperform the other competitive MOT methods in terms of RC, PRCS, MT, ML, FRG and IDS, which shows the significance and robustness of the proposed synthesizing of CRF matching and online hybrid boosting in associating tracklets. The instances of the tracking results using CRF-Boosting MOT are shown in Figure 7.

*Conclusions from Experimental Results on Different Datasets:* From the experimental results on different datasets, we could show the general outperformance of the proposed MOT approach on the CAVIAR dataset and its good performance compared to the other online MOT methods is also verified on the ETH dataset. However, from the results on the PETS dataset, we found that it may be required for the proposed MOT approach to adopt a motion pattern learning approach to improve MOT performance further through modelling nonlinear motion affinity. Also, as the other MOT methods, CRF-Boosting MOT also suffers from performance degradation problems for densely crowded and long-term occlusions. To remedy these issues, it would be beneficial to devise more an advanced appearance modelling approach (e.g., considering different poses and person re-identification module) and robust motion modelling approach (e.g., by learning different types of motion patterns).

*Discussion on Efficiency of CRF-Boosting Hybridization:* As we can easily can be seen from Table 6 that (i) ‘CRF-Boosting MOT w/o Boosting’ (i.e., only using CRF matching) produced the worst performance in terms of all metrics; (ii) ‘CRF-Boosting MOT w/o CRF Matching’ (i.e., only using online hybrid boosting) was slightly better than ‘CRF-Boosting MOT w/o Boosting’; and (iii) CRF-Boosting MOT (i.e., with CRF matching and online hybrid boosting) outperformed the others. From this, we can conclude that by synthesizing the two components together we could improve MOT performance.

*Discussion on Computational Speed:* We tested our proposed system on a PC equipped with an Intel^®^ Core™ i7-3770 CPU @ 3.40 GHz and 32 GB RAM, and the program was coded in Visual Studio Professional 2010 C++ without any parallel programming. As shown in Table 7, the tracking speed of our system is approximately 17 FPS on the image size of 400 × 300. This indicates that that the proposed online MOT system has high feasibility to be executed in real-time with reasonable tracking accuracy.

## 6. Conclusions and Future Research Agendas

We have presented an online hybrid data association method based on hybrid boosting employing CRF matching to facilitate robust online MOT systems. In the proposed approach, called CRF-boosting, for data association, learned CRF is used to construct reliable low-level tracklets and then they are used as the input of the hybrid boosting. Due to the synergetic cascaded learning procedure, CRF-boosting is capable of ensuring sufficient robustness with noisy detection results (i.e., without accurate ground truth information). Also, a hierarchical association framework is established to improve tracking accuracy. Experiments on public datasets show that the proposed approach could generally outperform the other competitive methods, from which we could naturally conclude that such a hybridized proposal is effective. We only demonstrated hierarchical association of simple features. Although the challenging hand-crafted features such as color similarity-based histograms of oriented gradients with the HSV color space [50] can be also adopted, we did not consolidate such computationally expensive features in this work considering the tracking speed. As a future work, we will further optimize the codes to get better performance in terms of MOT speed. Also, the challenging features will be also incorporated into the hierarchical feature association framework. Finally, we note that the study of substituting the data association scheme based on deep learning methodology is being carried out to obtain significant performance enhancement in terms of tracking accuracy.

## Figures and Tables

**Table 1:** List of Features.

Idx	Description
1:	Length of *T*_1_ (or *T*_2_)
2:	Number of detection responses in *T*_1_ (or *T*_2_)
3:	Number of detection response in *T*_1_ (or *T*_2_) divided by length of *T*_1_ (or *T*_2_)
4:	$\chi^{2}$ distance between color histograms of the tail part of *T*_1_ and the head part of *T*_2_
5:	Appearance(color, texture) consistency of the object in the interpolated trajectory between *T*_1_ and *T*_2_
6:	Number of miss detected frames in the gap between *T*_1_ and *T*_2_
7:	Number of frames occluded by other tracklets in the frame gap between *T*_1_ and *T*_2_
8:	Number of miss detected frames in the gap divided by the frame gap between *T*_1_ and *T*_2_
9:	Number of frames occluded in the gap divided by the frame gap between *T*_1_ and *T*_2_
10:	Estimated time from *T*_1_’s head to the nearest entry point.
11:	Estimated time from *T*_2_’s tail to the nearest exit point.
12:	Motion smoothness in image plane if *T*_1_ and *T*_2_ are linked
13:	Motion smoothness in ground plane if *T*_1_ and *T*_2_ are linked

**Table 2:** Evaluation Metrics.

Metric	Description
Ground Truth (**GT**)	Number of trajectories in the ground truth.
Recall (**RC**)	Number of correctly matched detections divided by the total number of detections in GT.
Mostly tracked trajectories (**MT**)	Percentage of trajectories that are successfully tracked for more than 80% divided by GT.
Partially tracked trajectories (**PT**)	Percentage of trajectories that are tracked between 20% and 80% divided by GT.
False alarm per frame (**FAF**)	Number of false alarms per frame
Mostly lost trajectories (**ML**)	Percentage of trajectories that are tracked for less than 20% divided by GT.
Fragments (**FRG**)	Total number of times that a trajectory in ground truth is interrupted by the tracking results.
ID switches (**IDS**)	Total number of times that a tracked trajectory changes its matched GT identity.

**Table 3:** Performance evaluation on CAVIAR.

Method	RC	PRCS	FAF	GT	MT	PT	ML	FRG	IDS
Wu and Nevatia [47]	75.2%		0.281	140	75.7%	17.9%	6.4%	35	17
Zhang et al. [7]	76.4%		0.105	140	85.7%	10.7%	3.6%	20	15
Huang et al. [39]	86.3%		0.186	143	78.3%	14.7%	7.0%	54	12
Li et al. [24]	89.0%		0.157	143	84.6%	14.0%	1.4%	17	11
Kuo et al. [37]	89.4%	96.9%	0.085	143	84.6%	14.7%	0.7%	18	11
Bak et al. [28]	-		-	-	84.6%	9.5%	5.9%	-	-
Yang et al. [48]	90.2%	96.1%	0.095	143	89.1%	10.2%	0.7%	11	5
CRF-Boosting MOT	93.1%	98.5%	0.099	143	86.7%	12.1%	1.2%	17	10

**Table 4:** Performance evaluation on PETS.

Method	RC	PRCS	FAF	GT	MT	PT	ML	FRG	IDS
Kuo et al. [22]	89.5%	99.6%	0.020	19	78.9%	21.1%	0.0%	23	1
Yang et al. [48]	91.8%	99.0%	0.053	19	89.5%	10.5%	0.0%	9	0
Chari et al. [13]	92.4%	94.3%	-	19	94.7%	5.3%	0.0%	74	56
Ba et al. [29]	90.2%	87.6%	-	-	-	-	-	-	-
Milan et al. [31]	92.4%	98.4%		23	91.3%	4.3%	4.4%	6	11
Milan et al. [25]	96.8%	94.1%	-	19	94.7%	5.3%	0.0%	15	22
Wen et al. [20]	93.3%	98.7%		23	95.7%	4.3%	0.0%	10	5
CRF-Boosting MOT	91.1%	99.2%	0.031	19	89.9%	10.1%	0.0%	10	0

**Table 5:** Performance evaluation on ETH.

Method	RC	PRCS	FAF	GT	MT	PT	ML	FRG	IDS
Kuo et al. [22]	76.8%	86.6%	0.891	125	58.4%	33.6%	8.0 %	23	11
Kim et al. [49]	78.4%	84.1%	0.977	124	62.7%	29.6%	7.7%	72	5
Bo and Nevatia [38]	79.0%	90.4%	0.637	125	68.0%	24.8%	7.2%	19	11
Milan et al. [25]	77.3%	87.2%	-	-	66.4%	25.4%	8.2%	69	57
Poiesi et al. [26]	78.7%	85.5%	-	125	62.4%	29.6%	8.0%	69	45
Bae and Yoon [27]	-		-	126	73.81%	23.81	2.38%	38	18
Ukita and Okada [30]	-		-	-	70.0%	25.2%	4.8%	30	17
CRF-Boosting MOT	79.1%	92.8%	0.805	125	81.3%	17.2%	1.5%	11	2

**Table 6:** Effects of CRF Matching and Online Hybrid Boosting.

Method	RC	PRCS	FAF	GT	MT	PT	ML	FRG	IDS
CRF-Boosting MOT w/o Boosting	87.3%	94.6%	0.203	143	80.3%	14.7%	5.0%	45	14
CRF-Boosting MOT w/o CRF-Matching	88.0%	95.0%	0.157	143	84.2%	13.6%	2.2%	17	11
CRF-Boosting MOT	93.1%	98.5%	0.099	143	86.7%	12.1%	1.2%	17	10

**Table 7:** Comparison of the Execution Time.

Method	Evaluation Speed	Conditions
Online Boosting-MOT [37]	Approx. 4 FPS	− Tested on CAVIAR dataset − Codes were implemented using Matlab
Online CRF-MOT [38]	Approx. 10 FPS	− Tested on ETH dataset − Codes were implemented using C++
CRF-Boosting MOT w/o Boosting	20.9 FPS	− Tested on CAVIAR dataset − Codes were implemented using C++
CRF-Boosting MOT w/o CRF-Matching	18.3 FPS	− Tested on CAVIAR dataset − Codes were implemented using C++
CRF-Boosting MOT	**17.4 FPS**	− Tested on CAVIAR dataset − Codes were implemented using C++
